# Distributed Optimal Power and Rate Control in Wireless Sensor Networks

**DOI:** 10.1155/2014/580854

**Published:** 2014-05-08

**Authors:** Meiqin Tang, Jianyong Bai, Jing Li, Yalin Xin

**Affiliations:** ^1^School of Mathematics and Statistics, Ludong University, Yantai 264025, China; ^2^Department of Automation and Electrical Engineering, Lanzhou Jiaotong University, Lanzhou 730070, China; ^3^School of Information Engineering, Nanchang University, Nanchang 330031, China

## Abstract

With the rapid development of wireless sensor networks, reducing energy consumption is becoming one of the important factors to extend node lifetime, and it is necessary to adjust the launching power of each node because of the limited energy available to the sensor nodes in the networks. This paper proposes a power and rate control model based on the network utility maximization (NUM) framework, where a weighting factor is used to reflect the influence degree of the sending power and transmission rate to the utility function. In real networks, nodes interfere with each other in the procedure of transmitting signal, which may lead to signal transmission failure and may negatively have impacts on networks throughput. Using dual decomposition techniques, the NUM problem is decomposed into two distributed subproblems, and then the conjugate gradient method is applied to solve the optimization problem with the calculation of the Hessian matrix and its inverse in order to guarantee fast convergence of the algorithm. The convergence proof is also provided in this paper. Numerical examples show that the proposed solution achieves significant throughput compared with exiting approaches.

## 1. Introduction

In recent years, with the consistent development of wireless sensor networks, they have been extensively applied in environmental monitoring, volcano monitoring, forest fire prevention, military facilities, and other fields [1]. A wireless sensor network is composed of a large number of microsensors which are capable of sensing, data processing, and transmission. It transmits signals through the self-organization form of networks and the cooperation among nodes. The energy of sensor nodes is very limited, and generally the battery of nodes is not non-renewable or its' updating is costlywhich results in an energy-constrained problem. Data transmission consumes most energy of sensor nodes. Therefore, how to control transmission power has become a key problem in the research of wireless sensor networks for low-energy consumption in data transmission under the premise of quality of services.

In wireless sensor networks, power control is to continuously adjust the transmission power of network nodes through a reasonable design. It can ensure the network connectivity and reduce the mutual interference among nodes to extend the lifetime of the whole sensor network. We consider the power and rate control problem based on the network utility framework, which has been introduced in wired [2, 3] and wireless networks [4–11] since the publication of the seminal paper [2] by Kelly et al. in 1998. In the past few years, a great deal of research effort has been made on the energy conservation in wireless sensor networks. One important perspective is to maximize the network lifetime [4, 5] while guaranteeing the required traffic rate. However, since sensor nodes are assumed to have fixed source rates, it is likely that the network cannot sustain the rate for the given system resource constraints. To this end, rate allocation in wireless sensor networks has been studied in recent years [6, 7]. In [6], the use of lexicographical max-min rate allocation was advocated for the nodes and a polynomial-time algorithm was developed for exploiting the parametric analysis technique from linear programming. The rate maximization problem in [7] was formulated as a concave utility maximization and a subgradient algorithm was proposed to solve it distributively. Game theory was applied to the power control problem based on the network utility maximization (NUM) framework in wireless sensor networks [8, 9]; the appropriate transmission power was selected to improve the network topology, reduce the collisions, increase good-put, and confirm the network connectivity. In [10], two algorithms were presented to compute the transmission power of each node with the objectives of minimizing the total transmission power and the total interference, respectively. But the time-varying wireless environment was not considered in the paper. The energy-constrained nature of nodes limits the operational lifetime of the network since energy is dissipated in both sensing and communicating data across the network. There is an intrinsic tradeoff between network lifetime maximization and rate allocation in wireless sensor networks. In [11], this tradeoff was characterized by considering a cross-layer design problem in a wireless sensor network with orthogonal link transmissions, and then the dual theory was used to solve the optimization problem. A priced-based distributed power and rate control algorithm was proposed in [12]; it can simulate the cooperation of power control and rate adaptation among the nodes. A joint source-channel maximum likelihood (SCML) decoding framework is proposed in wireless sensor networks (WSNs) [13], and prediction likelihood tree (PLT) approach is applied to exploit the spatiotemporal narrowband properties of the sensor data for sequence detection.

Considering the time-varying rate constraint, this paper presents a power and control algorithm based on the NUM framework which is similar to the algorithm proposed in [11, 12]. We adopt the probability to the constraint, which is more suitable for the varying wireless environment. The target function is decomposed into two optimization subproblems using dual decomposition methods to reduce the complexity of the solution for the large-scale network. We solve the optimization problem using the conjugate gradient method without the calculation of the Hessian matrix and its inverse. This can guarantee fast convergence of the algorithm.

The rest of the paper is organized as follows. The system model is described in Section 2. The proposed algorithm is formulated in Section 3. We give the convergence of the proposed algorithm in Section 3, numerical results are provided in Section 4, and Section 5 concludes.

## 2. Formulation of Power Optimization

### 2.1. System Description

Considering a wireless sensor network that consists of a set of *N* sensor nodes and a set of *L* wireless communication links, we assume that the link exists only when the nodes communicate directly. The transmission rate of node *s* is defined as *x*_*s*_, where *s* ∈ *N* and *x*_*s*_ ≥ 0 is satisfied. Let *p*_*s*_ denote the transmission power of node *s* with 0 ≤ *p*_*s*_ ≤ *p*_max⁡_, where *p*_max⁡_ is the maximum power. In the production and design of wireless sensor nodes, the survival time is generally designed to be not less than a constant value *T*_0_. Assuming that most of the energy is consumed in the process of signal transmission and the other part of the energy loss is ignored, the energy of wireless sensor network nodes is stored as a constant value *E*, and then *p*_max⁡_ is given by

(1) $$\begin{array}{c} p_{\max}=\frac{E}{T_{0}}. \end{array}$$

Assume there exists a link *l* (*l* ∈ *L*) which is connected with the other and can be modeled as a an additive white Gaussian noise (AWGN) channel with a noise spectral density *N*_0_. The channel capacity *c*_*l*_ of link *l* can be given by Shannons theory [14]:

(2) $$\begin{array}{c} c_{l}\left(p_{l}\right)=w\text{lo}{\text{g}}_{2}\left(1+\frac{p_{l}Kd_{l}^{-\alpha }}{N_{0}w}\right), \end{array}$$

where *w* is the fixed bandwidth, the transmission distance between the transmitter and receiver on link *l* is *d*_*l*_, *K* is a constant that depends on the transmission frequency, and *α* is the path-loss exponent, and therefore we have the following restriction condition:

(3) $$\begin{array}{c} \underset{s}{\sum }x_{s}\leq c_{l}\left(p_{l}\right). \end{array}$$

Since the node may produce mutual influence and interference when transmitting the signal, the transmission of signals between adjacent nodes is not always successful. *q*_*s*_ is assumed to be the probability of the successful signal transmission between adjacent nodes. We can give the following improved constraint condition:

(4) $$\begin{array}{c} \underset{s}{\sum }q_{s}x_{s}\leq c_{l}\left(p_{l}\right). \end{array}$$

Now we describe the calculating procedure of probability *q*_*s*_. The number of the nodes in link *l* which may compete with the node *s* is *n*_*s*_  (*n*_*s*_ ∈ *N*). In the case of competition, node *s* is to measure how much probability of *q*_*s*_ denotes the transmit success probability of node *s*. Given that the success transmitting gain is *u*_*s*_, which is related to the utilization ratio of energy for the node, the failure transmitting gain is *u*_*c*_ and the gain that the node does not transmit is *u*_*i*_. Obviously, we can get *u*_*c*_ < *u*_*i*_ < *u*_*s*_ since the utilization ratio of energy for the node is low when the node transmission fails, while the ratio is high when the node transmission succeeds. Consequently, the transmit success probability of node *s* is (1 − *q*_*s*_)^*n*_*s*_^, the transmission failure probability of node *s* is 1 − (1 − *q*_*s*_)^*n*_*s*_^, and the transmission signal gain *u*_*t*_ of node *s* can be given by

(5) $$\begin{array}{c} u_{t}={\left(1-q_{s}\right)}^{n_{s}}u_{s}+\left[1-{\left(1-q_{s}\right)}^{n_{s}}\right]u_{c}. \end{array}$$

When the node does not transmit the signal, the signal gain *u*_*w*_ is

(6) $$\begin{array}{c} u_{w}=u_{i}. \end{array}$$

Each node can choose to transmit the signal or not. There are two cases for transmission: success and failure. If one of the nodes successfully transmits the signal, the other nodes are all in transmission failure state or no transmission state. In order to prolong the lifetime of the whole system, the gain of the nodes needs to be balanced. Suppose *u*_*t*_ = *u*_*ω*_, and we get

(7) $$\begin{array}{c} q_{s}=1-{\left(\frac{u_{i}-u_{c}}{u_{s}-u_{c}}\right)}^{1/n_{s}\;}. \end{array}$$

We can find that the success transmission probability is related not only to the node number but also to the gain of the transmission success, transmission failure, and no transmission. The gains are always defined as

(8) $$\begin{array}{c} u_{s}=\omega_{1}p_{s}\log p_{s},\;\;u_{c}=\omega_{2}p_{s}\log p_{s},\;\;u_{i}=0, \end{array}$$

where *ω*_1_ and *ω*_2_ are constants. Submitting (8) into (7), we can get

(9) $$\begin{array}{c} q_{s}=1-{\left(\frac{w_{2}}{w_{2}-w_{1}}\right)}^{1/n_{s}\;}. \end{array}$$

The utility function *U*_*s*_ based on the NUM framework denotes the satisfaction degree of the user, which is continuously differentiable while increasing. Taking into account the transmission rate and transmission power, the total utility function based on the network utility function is defined as

(10) $$\begin{array}{c} \alpha \underset{s}{\sum }U_{s}^{1}\left(x_{s}\right)-\left(1-\alpha \right)\underset{l}{\sum }\underset{s}{\sum }U_{s}^{2}\left(p_{l}\right), \end{array}$$

where *α* is the weight which can reflect the influence degree of the utility function to the transmission power and rate, and it satisfies 0.05 ≤ *α* ≤ 0.95.

We can observe that a given encoding distortion can be guaranteed by controlling both the source rate and the encoding power. When simply adjusting the source rate or the encoding power to a very low or very high level, the encoding distortion will inevitably become large while the total power consumed at the sensor node will increase fast. In this paper, we consider the power and rate control problem with the power and rate constraints. The optimization problem for resource control is formulated as

(11) max⁡    α∑sUs1(xs)−(1−α)∑l∑sUs2(pl)s.t.     ∑sqsxs≤cl(pl), l=1,2,…,L0≤pl≤pmax⁡0.05≤α≤0.95,xs≥0.

### 2.2. Dual Decomposition of the Proposed Algorithm

Two Lagrange multipliers *λ*_*l*_ and *μ*_*l*_ are introduced to the Lagrangian dual function of the primal problem (11), and the corresponding Lagrangian dual function can be expressed as

(12) L(xs,pl,λl,μl) =α∑sUs1(xs)−(1−α)∑l∑sUs2(pl)  +∑lλl[cl(pl)−∑sqsxs]+∑lμl(pmax⁡−pl) =∑s[αUs1(xs)−(1−α)∑lUs2(pl)−qsxs∑lλl]  +∑lλlcl(pl)+∑lμl(pmax⁡−pl).

The dual problem is then given by

(13) min⁡        D(λ,μ) s.t.           λ≥0, μ≥0.

We can get the dual function as follows:

(14) D(λ,μ) =max⁡∑s[αUs1(xs)−(1−α)∑lUs2(pl)−qsxs∑lλl]  +∑l[λlcl(pl)+μl(pmax⁡−pl)].

The dual function can be decomposed into two subproblems, which are evaluated separately for the nodes, and then the dual function can be rewritten as

(15) D(λ,μ)=max⁡αUs1(xs)−(1−α)∑lUs2(pl) −qsxs∑lλl+λscl(ps)+μs(ps max⁡−ps).

The subproblem of optimization with *x*_*s*_ as the variable is

(16) max⁡     αUs1(xs)−qsxs∑lλl s.t.     xs≥0.

The subproblem of optimization with *p*_*l*_ as the variable is

(17) max⁡    −(1−α)∑lUs2(pl)+λlcl(pl)+μl(pmax⁡−pl) s.t.      0≤pl≤pmax⁡.

According to the dual theory, we can get the following equation for the rate suboptimization problem from (16):

(18) min⁡    qsxs∑lλl−αUs1(xs) s.t.     xs≥0.

And the power suboptimization problem as described in (17) can be rewritten as

(19) min⁡    (1−α)∑lUs2(pl)−λlcl(pl)−μl(pmax⁡−pl) s.t.     0≤pl≤pmax⁡,

where *f*(*x*_*s*_) and *f*(*p*_*l*_) are twice continuously differentiable. We used the conjugate gradient method mentioned before to calculate, which has many advantages. It only needs to seek the function of the first order derivative, which not only alleviates the slow convergence characteristic of the steepest descent method, but also avoids the storage and computation of Hessian matrix and its inverse features in Newton's method. Besides, the program compilation is relatively simple and the computational complexity is relatively small. It is the most effective solution of a large number of linear equations and nonlinear unconstrained optimization problems.

For (16), we obtain the guiding function through the derivation of *λ*_*l*_, *μ*_*l*_:

(20) $$\begin{array}{c} \frac{\partial }{\partial \lambda_{l}}\left(\left(1-\alpha \right)U_{s}^{1}\left(x_{s}\right)-q_{s}x_{s}\underset{l}{\sum }\lambda_{l}\right)=-q_{s}x_{s}. \end{array}$$

For (17), we get the guiding function through the derivation of *λ*_*l*_, *μ*_*l*_:

(21) ∂∂λl((α−1)∑lUs2(pl)+λlcl(pl)+μl(pmax⁡−pl))  =cl(pl)

(22) ∂∂μl((α−1)∑lUs2(pl)+λlcl(pl)+μl(plmax⁡−pl))  =plmax⁡−pl.

According to (20) and (21), the step iterative algorithm of *λ*_*l*_ is

(23) $$\begin{array}{c} \lambda_{l}\left(k+1\right)={\left[\lambda_{l}\left(k\right)+\gamma \left(k\right)\left[c_{l}\left(p_{l}\right)-q_{s}x_{s}\right]\right]}^{+}. \end{array}$$

According to (22), the step iterative algorithm of *μ*_*l*_ is

(24) $$\begin{array}{c} \mu_{l}\left(k+1\right)={\left[\mu_{l}\left(k\right)+\kappa \left(k\right)\left(p_{\max}-p_{l}\right)\right]}^{+}, \end{array}$$

where *γ*(*k*) and *κ*(*k*) are the step length.

The principle and the procedure of power *p*_*l*_ and rate *x*_*s*_ optimal algorithm are similar, which are all optimized based on the conjugate gradient method. And we used decomposition theory to decompose the two algorithms into one separately and they are connected by the Lagrange multipliers to be combined to one problem to assure the total utility is to be maximized. The steps of the optimization algorithm based on the conjugate gradient method for *x*_*s*_ are as follows.

**Step 1**

Given an initial value of *p*_*l*_(0), *μ*_*l*_(0), and *μ*_*l*_(0), where the error *ɛ* > 0, calculate *f*(0) as follows:

(25) $$\begin{array}{c} f\left(0\right)=f\left(p_{l}\left(0\right)\right), \end{array}$$

and then set

(26) $$\begin{array}{c} d\left(0\right)=-\Delta f\left(p_{l}\left(0\right)\right),\;k=0. \end{array}$$

**Step 2**

If

(27) $$\begin{array}{c} ||f\left(p_{l}\left(k\right)\right)||\leq ɛ \end{array}$$

is satisfied, stop the calculation; otherwise, go to Step 3.

**Step 3**

Calculate step length factor *ζ*_*k*_ through the linear search method, where *ζ*_*k*_ satisfies the strong Wolfe linear search criteria:

(28) $$\begin{array}{c} f\left(p_{l}\left(k\right)\right)+\zeta_{k}d_{k}=\min_{\zeta >0}f\left(p_{l}\left(k\right)+\zeta \left(k\right)d\left(k\right)\right). \end{array}$$

Assume

(29) $$\begin{array}{c} p_{l}\left(k+1\right)=p_{l}\left(k\right)+\zeta_{k}d_{k},\\ \lambda_{l}\left(k+1\right)={\left[\lambda_{l}\left(k\right)+\gamma \left(k\right)\left[c_{l}\left(p_{l}\right)-q_{s}x_{s}\right]\right]}^{+},\\ \mu_{l}\left(k+1\right)={\left[\mu_{l}\left(k\right)+\kappa \left(k\right)\left(p_{\max}-p_{l}\right)\right]}^{+}. \end{array}$$

**Step 4**

Calculate

(30) $$\begin{array}{c} \chi_{k}=\frac{{||\nabla f\left(p_{l}\left(k+1\right)\right)||}^{2}}{{||\nabla f\left(p_{l}\left(k\right)\right)||}^{2}},\\ d_{k+1}=-\nabla f\left(p_{l}\left(k+1\right)\right)+\chi_{k}d_{k}. \end{array}$$

**Step 5**

Consider

(31) $$\begin{array}{c} k=k+1; \end{array}$$

then, go to Step 2.

In the algorithm, we must guarantee the direction is decreased, which requires

(32) (∇f(pl(k)),d(k))=(∇f(pl(k)),−∇f(pl(k)))=−||∇f(pl(k))||2<0.

So we use the strong Wolfe linear search criteria to calculate the step length factor *ζ*_*k*_, and the direction of *d*_*k*_ is definitely the descent direction.

The steps of the optimization algorithm based on the conjugate gradient method for *x*_*s*_ are similar to *p*_*l*_.

**Step 1**

Given an initial value of *x*_*s*_(0), *μ*_*l*_(0), and *μ*_*l*_(0), where the error *ɛ*′ > 0, calculate *f*′(0):

(33) $$\begin{array}{c} f^{\prime }\left(0\right)=f^{\prime }\left(p_{l}\left(0\right)\right), \end{array}$$

and then set

(34) $$\begin{array}{c} d^{\prime }\left(0\right)=-\Delta f\left(p_{l}\left(0\right)\right),\;k^{\prime }=0. \end{array}$$

**Step 2**

If

(35) $$\begin{array}{c} ||f^{\prime }\left(p_{l}\left(k\right)\right)||\leq {ɛ}^{\prime } \end{array}$$

is satisfied, stop the calculation; otherwise, go to Step 3.

**Step 3**

Calculate the step length factor *ζ*_*k*_′ through the linear search method, where *ζ*_*k*_′ satisfies the strong Wolfe linear search criteria:

(36) $$\begin{array}{c} f^{\prime }\left(x_{s}\left(k\right)\right)+\zeta_{k}^{\prime }d_{k}^{\prime }=\underset{\zeta^{\prime }>0}{\min}f^{\prime }\left(x_{s}\left(k\right)+\zeta^{\prime }\left(k\right)d^{\prime }\left(k\right)\right). \end{array}$$

Assume

(37) $$\begin{array}{c} x_{s}\left(k+1\right)=x_{s}\left(k\right)+\zeta_{k}^{\prime }d_{k}^{\prime },\\ \lambda_{l}\left(k+1\right)={\left[\lambda_{l}\left(k\right)+\gamma \left(k\right)\left[c_{l}\left(p_{l}\right)-q_{s}x_{s}\right]\right]}^{+},\\ \mu_{l}\left(k+1\right)={\left[\mu_{l}\left(k\right)+\kappa \left(k\right)\left(p_{\max}-p_{l}\right)\right]}^{+}. \end{array}$$

**Step 4**

Calculate

(38) $$\begin{array}{c} \chi_{k}^{\prime }=\frac{{||\nabla f^{\prime }\left(x_{s}\left(k+1\right)\right)||}^{2}}{{||\nabla f^{\prime }\left(x_{s}\left(k\right)\right)||}^{2}},\\ d_{k+1}^{\prime }=-\nabla f^{\prime }\left(p_{l}\left(k+1\right)\right)+\chi_{k}^{\prime }d_{k}^{\prime }. \end{array}$$

**Step 5**

Consider

(39) $$\begin{array}{c} k=k+1, \end{array}$$

and then go to Step 2.

In the algorithm, we must guarantee the direction is decreased, which requires

(40) (∇f′(xs(k)),d(k))=(∇f′(xs(k)),−∇f′(xs(k)))=−||∇f′(xs(k))||2<0.

## 3. Convergence Analyses

For the general function, the conjugate gradient method under certain conditions is convergent, and the convergence speed is generally superior to that of the steepest descent method. The *p*_*l*_'s convergence will be proved as follows. It is similar to *x*_*s*_.

**Proposition 1**

Assume that *f*(*p*_*l*_) is in the bounded set

(41) $$\begin{array}{c} L=\left\{p_{l}\in R\;|\;f\left(p_{l}\right)\leq f\left(p_{l}\left(0\right)\right)\right\}, \end{array}$$

which is continuously differentiable and has a lower bound; then, the sequence obtained by the conjugate gradient method {*p*_*l*_} converges to *p*_l_*, which is the stagnation point of *f*(*p*_*l*_).

**Proof**

Given that {*p*_*l*_(*k*)} is a finite sequence of number, according to the algorithms termination conditions, the last *p*_l_* must meet

(42) $$\begin{array}{c} g\left(p_{l}^{*}\right)=0, \end{array}$$

so *p*_l_* is the stagnation point of *f*(*p*_*l*_).

If {*p*_*l*_(*k*)} is an infinite series, then for all *k*,

(43) $$\begin{array}{c} \nabla f\left(p_{l}\left(k\right)\right)\neq 0. \end{array}$$

Since

(44) $$\begin{array}{c} d_{k}=-\nabla f\left(p_{l}\left(k\right)\right)+\chi_{k}d_{k-1}, \end{array}$$

we get

(45) $$\begin{array}{c} \left(\nabla f\left(p_{l}\left(k\right)\right),d_{k}\right)=-{||\nabla f\left(p_{l}\left(k\right)\right)||}^{2}<0, \end{array}$$

where *d*_*k*_ is the decline direction. Since {*f*(*p*_*l*_(*k*))} is the lower bound of the sequence and is monotonically decreasing,

(46) $$\begin{array}{c} f\left(p_{l}\left(k\right)\right)⟶f\left(p_{l}^{*}\right). \end{array}$$

The convergence analysis of *x*_s_* is similar to *p*_l_*.

## 4. Numerical Example

We simulate a network which consists of nine sensor nodes and one sink node. The sensor nodes will transmit their sensing data to the sink node, and all the nodes are randomly deployed in an area of 100 m × 100 m. The utility function is set to be in the log⁡ form; for example, *U*1 is set to be log⁡2(*x*) and *U*2 = −*p*. The fixed bandwidth *w* is set to 5 MHz and the maximum of *p* is set to be 1 mW for all links. The path-loss exponent *σ* is set to 2. We will show the network performance with different *α* values.

**Figure 1:**
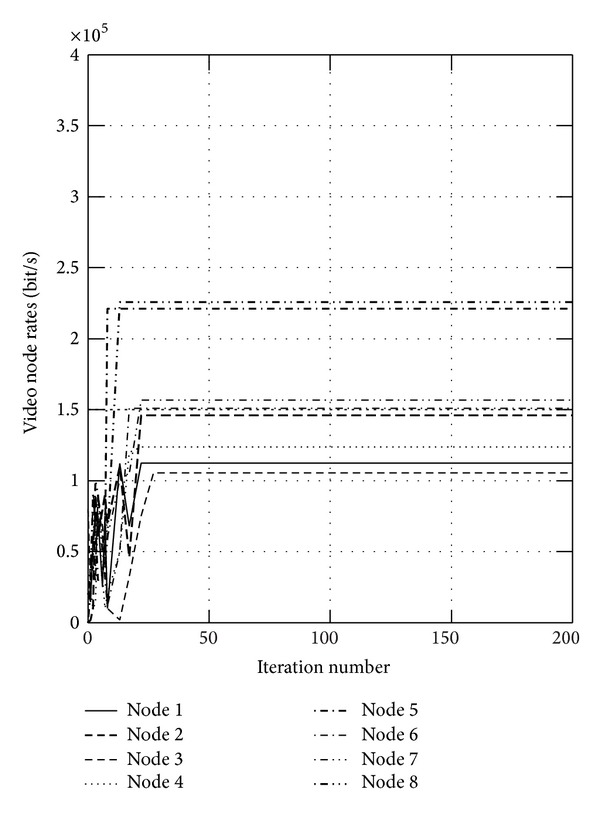
The optimal video rates for sensor nodes when *α* is set to 0.05.

**Figure 2:**
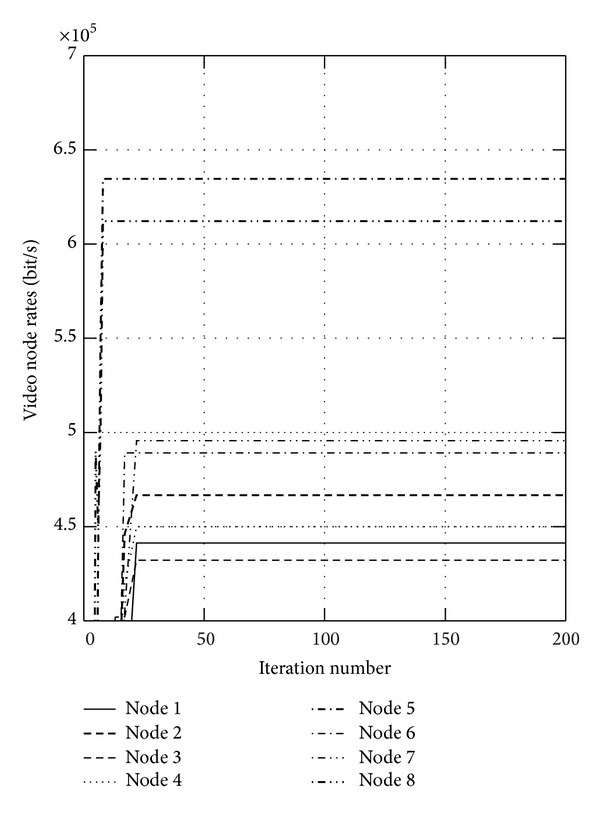
The optimal video rates for sensor nodes when *α* is set to 0.95.

**Figure 3:**
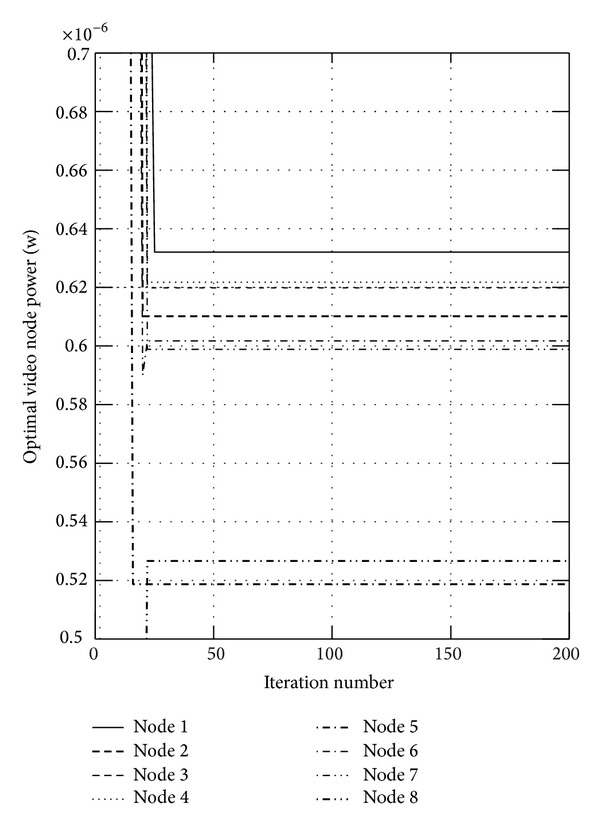
The consumed video power of the nodes when *α* is set to 0.05.

**Figure 4:**
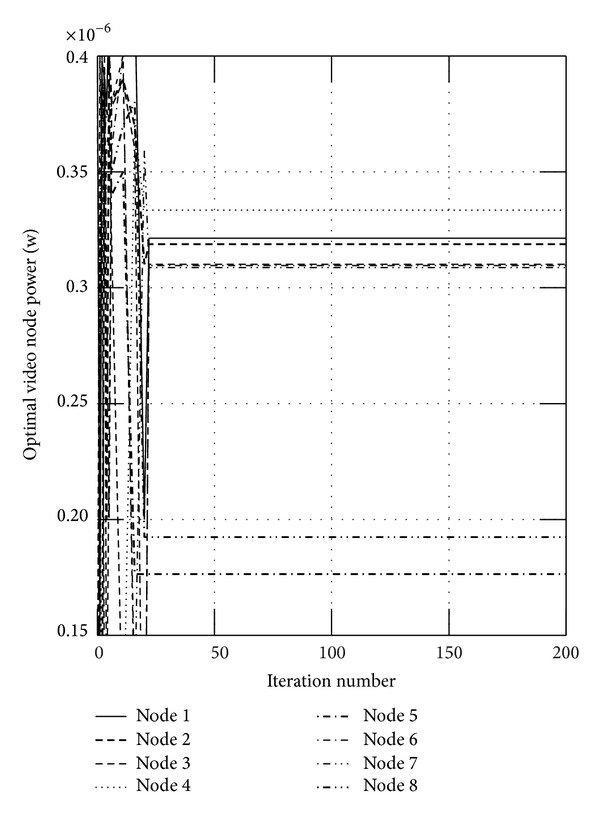
The consumed video power of the nodes when *α* is set to 0.95.

First, we show the convergence figures for the optimal rates and power. The maximum iteration of the algorithm is set to be 200. Figures 1 and 2 show the optimal video rates for sensor nodes when *α* is set to 0.05 and 0.95, respectively, from which we can find that the proposed algorithm can converge within 20 iteration steps. We also find the located rates of some nodes are large, while the others are small. This is because these nodes transmit the data to the sink node directly, which will not relay the data and will transmit the data more efficiently. In wireless sensor networks, the available energy is limited, and Figures 3 and 4 show the consumed video power of the nodes, from which we can also indicate the proposed algorithm can get to the optimum efficiently and quickly. From these four figures, we can find that the proposed algorithm can get higher throughput and uses less energy.

**Figure 5:**
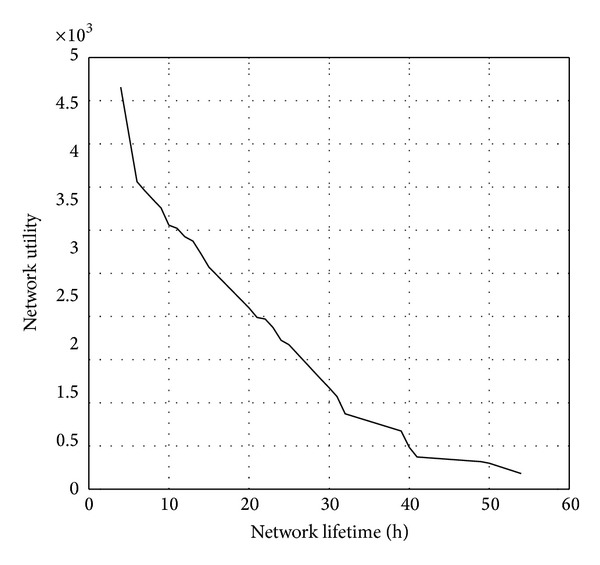
The tradeoff between network utility and network lifetime.

In Figure 5, we find that when we set *α* to 0.95, the utility is large, but the value of lifetime is small; when the *α* is set to 0.05, the value of lifetime is large, while the network utility is small. For this reason, we can find that there is apparently a tradeoff between network utility and network lifetime in energy-limited wireless networks. Different values can be set according to actual need.

In order to verify the performance of the proposed algorithm, we compared it with previous works in [11, 12], and the network setup is the same. Tables 1 and 2 give the data rate comparison with different values. From these two tables we can find that the proposed algorithm can get higher utility than the algorithm in [12] and minUtility [11] and less power consumption than the algorithm in [12] and MaxUtility [11], because minUtility only seeks minimum energy consumption and while MaxUtility only seeks the maximum utility. Through adding the probability for the rate constraint, our algorithm can get higher data rate.

Tables 3 and 4 show the power of the different algorithms, for which we can see that the proposed algorithm get less power consumption than the algorithm proposed in [12] and MaxUtility [11], which shows the proposed algorithm is very efficiently.

## 5. Conclusion

This paper proposes a new power control method based on network utility maximization framework for wireless sensor networks, and a trade-off parameter for the utility and lifetime is introduced to the system. Considering the time varying environment, the probability is added to the rate constraint, which is natural in the context of various applications. Moreover, we put forward a new priced-based distributed algorithm using a gradient method. The algorithm is designed to keep acceptable throughput. Simulation studies show that the proposed algorithms are effective to solve the optimization problem and outperform the existing approaches in terms of throughput and energy efficiency since desired variables converge to the optimal point very quickly.

## Figures and Tables

**Table 1:** The data rate comparison with different methods for *α* = 0.05.

Method	Node 1	Node 2	Node 3	Node 4	Node 5	Node 6	Node 7	Node 8
The proposed algorithm	1.1256*e* + 05	1.4621*e* + 05	1.0543*e* + 05	1.2390*e* + 05	2.2111*e* + 05	1.5098*e* + 05	1.5678*e* + 05	2.2567*e* + 05
The algorithm in [12]	1.1021*e* + 05	1.3609*e* + 05	0.9763*e* + 05	1.1907*e* + 05	1.8798*e* + 05	1.3142*e* + 05	1.3988*e* + 05	2.0995*e* + 05
MaxUtility [11]	1.26*e* + 05	1.4569*e* + 05	1.1314*e* + 05	1.3086*e* + 05	1.9209*e* + 05	1.7142*e* + 05	1.6678*e* + 05	1.8765*e* + 05
MinEnergy [11]	92	89.21	98.74	99.2356	101.48	99.672	96.32	102.54

**Table 2:** The data rate comparison with different methods for *α* = 0.95.

Method	Node 1	Node 2	Node 3	Node 4	Node 5	Node 6	Node 7	Node 8
The proposed algorithm	4.4123*e* + 05	4.6678*e* + 05	4.321*e* + 05	4.5009*e* + 05	6.3457*e* + 05	4.8914*e* + 05	4.9562*e* + 05	6.1209*e* + 05
The algorithm in [12]	4.2265*e* + 05	4.4324*e* + 03*e* + 05	4.5616*e* + 05	4.3190*e* + 05	4.4999*e* + 05	6.2008*e* + 05	4.81998*e* + 05	4.785*e* + 05
MaxUtility [11]	4.5532*e* + 05	4.7121*e* + 03*e* + 05	4.35143*e* + 05	4.590*e* + 05	4.8654*e* + 05	4.2672*e* + 05	4.996*e* + 05	4.9867*e* + 05
MinEnergy [11]	104.56	105.32	102.78	103.45	128.9	107.8	103.81	127.65

**Table 3:** The data power comparison with different methods for *α* = 0.05.

Method	Node 1	Node 2	Node 3	Node 4	Node 5	Node 6	Node 7	Node 8
The proposed algorithm	0.6321*e − *6	0.6102*e − *6	0.6198*e − *6	0.6218*e − *6	0.5187*e − *6	0.6018*e − *6	0.5989*e − *6	0.5266*e − *6
The algorithm in [12]	0.7112*e − *6	0.7426*e* + 03*e − *6	0.7002*e − *6	0.7221*e − *6	0.6789*e − *6	0.7108*e − *6	0.7685*e − *6	0.6351*e − *6
MaxUtility [11]	0.9168*e − *05	0.9041*e − *05	0.9908*e − *05	0.9127*e − *05	0.8873*e − *05	0.9162*e − *05	0.9092*e − *05	0.8904*e − *05
MinEnergy [11]	0.8465*e − *09	0.8169*e − *09	0.83287*e − *09	0.8658*e − *09	0.1099*e − *10	0.8365*e − *09	0.8102*e − *09	0.1214*e − *10

**Table 4:** The data rate comparison with different methods for *α* = 0.95.

Method	Node 1	Node 2	Node 3	Node 4	Node 5	Node 6	Node 7	Node 8
The proposed algorithm	0.3214*e* − 6	0.3189*e* − 6	0.3102*e* − 6	0.3336*e* − 6	0.1765*e* − 6	0.3087*e* − 6	0.3096*e* − 6	0.1924*e* − 6
The algorithm in [12]	0.4219*e* − 6	0.4468*e* + 03*e* − 6	0.4097*e* − 6	0.4354*e* − 6	0.2176*e* − 6	0.4677*e* − 6	0.4781*e* − 6	0.2674*e* − 6
MaxUtility [11]	0.5532*e* − 05	0.5096*e* − 05	0.5989*e* − 05	0.5213*e* − 05	0.3786*e* − 05	0.5011*e* − 05	0.5124*e* − 05	0.3989*e* − 05
MinEnergy [11]	0.4107*e* − 09	0.4231*e* − 09	0.4358*e* − 09	0.4691*e* − 09	0.0519*e* − 10	0.4218*e* − 09	0.449*e* − 09	0.0627*e* − 10
